# Supplementary material for: A downsampling strategy to assess the predictive value of radiomic features
Source: Sci Rep. 2019 Nov 28;9:17869. doi: 10.1038/s41598-019-54190-2 (PMC6883070; doi:10.1038/s41598-019-54190-2)
Supplement: Supplementary file 1 — Supplementary data [file 41598_2019_54190_MOESM1_ESM.pdf]

A downsampling strategy to assess the predictive value of radiomic features

Anne-Sophie Dirand[a]*, Frédérique Frouin[a], Irène Buvat[a]

[a] Imagerie Moléculaire In Vivo, CEA-SHJF, Inserm, CNRS, Université Paris-Sud, Université Paris-Saclay, Orsay, France

*Corresponding author.
E-mail address: dirandannesophie@gmail.com

Set-up	Training set + Validation set (TRS+VS)		Testing set (TES)		Number of folds for the SKFCV
	BM	MM	BM	MM	
S1	17	10	71	42	2
S2	34	20	71	42	2
S3	51	30	71	42	3
S4	68	40	71	42	4
S5	85	50	71	42	5
S6	102	60	71	42	6
S7	119	70	71	42	7
S8	136	80	71	42	8
S9	270	160	71	42	16
**S10**	**10**	**10**	**71**	**42**	**2**
**S11**	**20**	**20**	**71**	**42**	**2**
**S12**	**30**	**30**	**71**	**42**	**3**
**S13**	**40**	**40**	**71**	**42**	**4**
**S14**	**50**	**50**	**71**	**42**	**5**
**S15**	**60**	**60**	**71**	**42**	**6**
**S16**	**70**	**70**	**71**	**42**	**7**
**S17**	**80**	**80**	**71**	**42**	**8**
**S18**	**160**	**160**	**71**	**42**	**16**

Table S1 : Number of patients for each set-up of cohort 2 and synthetic cohort 2. Balanced situations in bold characters.

Set-up	Number of patients (TRS+VS)	A = number of features with Y_{ROC}^{TRS+VS} > 0.20	Number of features with Y_{ROC}^{TES} >0.20	B = Number of features selected on both TRS+VS and ETS (Youden index > 0.20)	$\frac{B}{A}$
S1	30PT 10MT	43	8	8	0.19
S2	60PT 20MT	42	13	13	0.31
S3	90PT 30MT	25	12	12	0.48
S4	120PT 40MT	21	14	14	0.67
S5	150PT 50MT	21	18	18	0.86
S6	180PT 60MT	21	13	13	0.62
S7	210PT 70MT	21	11	11	0.52
S8	240PT 80MT	21	13	13	0.62
S9	270PT 90MT	21	19	18	0.86
S10	30PT 30MT	40	10	10	0.25
S11	45PT 45MT	24	11	11	0.46
S12	60PT 60MT	21	14	14	0.67
S13	75PT 75MT	21	13	13	0.62
S14	90PL 90MT	21	16	16	0.76

Table S2 : Number of features that yielded a Youden index >0.20 for each experimental condition for cohort 1

Set-up	Number of patients (TRS+VS)	A = number of features with Y_{ROC}^{TRS+VS} > 0.75	Number of features with Y_{ROC}^{TES} >0.75	B = Number of features selected on both TRS+VS and ETS (Youden index > 0.75)	$\frac{B}{A}$
S1	10MM 17BM	11	4	4	0.36
S2	20MM 35BM	11	5	5	0.45
S3	30MM 50BM	9	5	5	0.56
S4	40MM 70BM	9	5	5	0.56
S5	50MM 80BM	10	5	5	0.5
S6	60MM 105BM	9	5	5	0.56
S7	70MM 117BM	7	5	5	0.71
S8	80MM 140BM	7	5	5	0.71
S9	160MM 280BM	7	5	5	0.71
S10	10MM 10BM	11	5	5	0.45
S11	20MM 20BM	11	5	5	0.45
S12	30MM 30BM	11	5	5	0.45
S13	40MM 40BM	11	5	5	0.45
S14	50MM 50BM	10	5	5	0.5
S15	60MM 60BM	9	5	5	0.56
S16	70MM 70BM	10	5	5	0.5
S17	80MM 80BM	7	5	5	0.71
S18	160MM 160BM	7	5	5	0.71

Table S3 : Number of features that yielded a Youden index>0.75 for each experimental condition for cohort 2.

Unbalanced set-ups	S1	S2	S3	S4	S5	S6	S7	S8	S9
$Y_{i,C_m}^{TES} \in Y_{i,C_m}^{VS} \pm SY_{i,C_m}^{VS}$	55	48	65	66	75	77	76	79	80

Balanced set-ups	S10	S11	S12	S13	S14	S15	S16	S17	S18
$Y_{i,C_m}^{TES} \in Y_{i,C_m}^{VS} \pm SY_{i,C_m}^{VS}$	59	51	70	71	74	82	81	86	93

Table S4 : For each set-up, percentage over 450 cases for which Y_{i,C_m}^{TES} *was included in* $Y_{i,C_m}^{VS} \pm SY_{i,C_m}^{VS}$ *(cohort 2).*

Number of patients	LR		RFELR		PCALR		ROCLR		SVM		RFESVM		PCASVM		ROCSVM		LASSO	
	QF1’	QF2’	QF1’	QF2’	QF1’	QF2’	QF1’	QF2’	QF1’	QF2’	QF1’	QF2’	QF1’	QF2’	QF1’	QF2’	QF1’	QF2’
220	0.01	0.02	0.01	0.02	0.02	0.02	0.02	0.02	0.01	0.02	0.02	0.02	***0.03***	0.01	0.02	0.03	0.02	0.04
212	0.01	0.02	0.01	0.03	0.00	0.02	0.01	0.02	0.01	0.03	0.02	0.02	***0.03***	0.01	0.00	0.03	0.02	0.04
204	0.01	0.02	0.00	0.03	0.01	0.01	0.02	0.02	0.03	0.03	0.01	0.02	***0.03***	0.01	0.01	0.03	0.03	0.04
196	0.01	0.02	0.00	0.03	0.01	0.02	0.02	0.02	0.03	0.03	0.01	0.03	***0.04***	0.01	***0.04***	0.03	0.03	0.05
188	0.01	0.02	0.01	0.04	0.02	0.02	0.02	0.02	***0.03***	0.02	0.01	0.03	***0.03***	0.01	***0.04***	0.03	0.02	0.04
180	0.02	0.03	0.01	0.04	0.01	0.02	0.01	0.03	0.02	0.03	0.02	0.03	***0.03***	0.01	0.*01*	0.04	0.04	0.04
172	0.01	0.02	0.01	0.03	0.01	0.02	0.02	0.02	0.03	0.03	0.01	0.02	***0.05***	0.02	***0.04***	0.03	0.03	0.05
164	0.01	0.03	0.01	0.05	0.01	0.03	0.01	0.02	***0.04***	0.03	0.02	0.03	***0.04***	0.02	0.03	0.03	0.02	0.05
156	0.00	0.04	0.01	0.05	0.02	0.04	0.01	0.03	0.04	0.04	0.01	0.05	***0.04***	0.02	***0.08***	0.05	0.01	0.05
148	0.01	0.03	0.00	0.05	0.01	0.04	0.02	0.03	***0.08***	0.04	0.03	0.04	***0.04***	0.03	***0.05***	0.04	0.02	0.07
140	0.01	0.04	0.02	0.05	0.00	0.04	0.03	0.04	0.05	0.06	0.02	0.05	***0.07***	0.02	***0.07***	0.05	0.01	0.07
132	0.01	0.03	0.02	0.07	0.01	0.04	0.02	0.04	0.05	0.06	0.04	0.05	***0.05***	0.04	0.05	0.05	***0.08***	0.06
124	0.00	0.04	0.03	0.06	0.00	0.05	0.02	0.02	***0.08***	0.06	0.01	0.06	***0.08***	0.05	0.04	0.04	0.02	0.07
116	0.01	0.08	0.04	0.10	0.01	0.08	0.00	0.04	0.03	0.08	0.01	0.08	0.06	0.06	***0.06***	0.05	0.01	0.10
108	0.03	0.10	0.03	0.11	0.03	0.09	0.00	0.06	0.05	0.08	0.02	0.09	0.05	0.06	***0.09***	0.07	0.03	0.11
100	0.02	0.08	0.02	0.09	0.02	0.08	0.01	0.07	0.08	0.11	0.01	0.09	***0.12***	0.06	***0.09***	0.07	0.00	0.10
92	0.00	0.08	0.05	0.11	0.01	0.11	0.00	0.08	0.08	0.10	0.07	0.11	***0.12***	0.06	0.09	0.10	0.01	0.10
84	0.02	0.10	0.01	0.12	0.00	0.10	0.03	0.10	0.08	0.11	0.02	0.11	***0.12***	0.10	0.09	0.14	0.01	0.12
76	0.04	0.09	0.03	0.13	0.00	0.10	0.02	0.10	0.08	0.14	0.02	0.12	***0.12***	0.09	0.09	0.13	0.00	0.15
68	0.04	0.12	0.04	0.16	0.03	0.11	0.01	0.09	0.08	0.16	0.07	0.14	***0.12***	0.11	0.09	0.16	0.01	0.15
60	0.04	0.13	0.02	0.13	0.04	0.11	0.01	0.12	0.08	0.17	0.07	0.13	0.12	0.15	0.09	0.16	0.04	0.17
52	0.06	0.15	0.02	0.16	0.07	0.14	0.00	0.10	0.08	0.20	0.07	0.14	0.12	0.15	0.09	0.15	0.05	0.17
44	0.04	0.16	0.07	0.18	0.02	0.18	0.03	0.15	0.08	0.25	0.02	0.22	0.12	0.19	0.09	0.24	0.03	0.19
36	0.17	0.19	0.01	0.20	0.10	0.18	0.05	0.19	0.08	0.30	0.07	0.22	0.12	0.27	0.09	0.28	0.02	0.21

Table S5 : QF for AUC for each convergence reaching classifier for each experimental condition (cohort 1). Bold and italic values: QF1’≥ QF2’.

Number of patients	LR		RFELR		PCALR		ROCLR		SVM		RFESVM		PCASVM		ROCSVM		LASSO	
	QF1’	QF2’	QF1’	QF2’	QF1’	QF2’	QF1’	QF2’	QF1’	QF2’	QF1’	QF2’	QF1’	QF2’	QF1’	QF2’	QF1’	QF2’
73	***0.03***	0.00	0.02	0.04	0.01	0.06	***0.07***	0.06	0.01	0.07	0.01	0.06	0.00	0.06	0.05	0.07	0.02	0.07
70	***0.03***	0.02	0.01	0.06	0.01	0.05	0.05	0.06	0.02	0.05	0.03	0.08	0.02	0.07	0.08	0.08	0.02	0.08
67	0.01	0.01	0.02	0.05	0.01	0.04	0.02	0.07	0.01	0.07	0.04	0.1	0.02	0.07	0.01	0.06	0.04	0.09
64	***0.06***	0.02	0.00	0.04	0.01	0.06	0.01	0.06	0.02	0.06	0.02	0.08	0.03	0.08	0.03	0.08	0.02	0.06
61	***0.02***	0.01	0.00	0.04	0.02	0.06	0.04	0.05	0.03	0.08	0.00	0.09	0.04	0.04	0.03	0.1	0.03	0.07
58	0.03	0.04	0.00	0.05	0.03	0.06	0.02	0.05	0.01	0.06	0.01	0.09	0.03	0.06	0.04	0.06	0.03	0.08
55	0.02	0.03	0.02	0.04	0.04	0.06	***0.04***	0.03	0.02	0.06	0.03	0.08	0.01	0.05	0.02	0.06	0.03	0.08
52	***0.05***	0.02	0.01	0.05	0.04	0.07	0.06	0.07	0.02	0.09	0.01	0.1	0.00	0.08	0.03	0.09	0.01	0.09
49	0.01	0.02	0.02	0.07	0.05	0.09	***0.07***	0.05	0.01	0.11	0.06	0.12	0.00	0.11	0.00	0.09	0.03	0.11
46	0.02	0.05	0.01	0.07	0.05	0.08	0.01	0.04	0.02	0.09	0.05	0.1	0.01	0.09	0.02	0.11	0.02	0.11
43	0.01	0.04	0.02	0.08	0.05	0.1	***0.07***	0.06	0.03	0.07	0.05	0.12	0.00	0.11	0.01	0.07	0.04	0.11
40	0.02	0.05	0.03	0.06	0.04	0.09	***0.06***	0.03	0.03	0.1	0.05	0.11	0.01	0.14	***0.12***	0.09	0.08	0.13
37	0.01	0.04	0.00	0.09	0.04	0.09	0.02	0.04	0.04	0.09	0.00	0.08	0.02	0.11	0.03	0.08	0.05	0.14
34	0.06	0.06	0.02	0.07	0.05	0.1	***0.08***	0.07	***0.12***	0.1	0.02	0.07	0.01	0.12	0.1	0.1	0.09	0.2
31	0.00	0.05	0.01	0.09	0.05	0.1	0.06	0.07	0.02	0.09	0.04	0.11	0.02	0.14	0.05	0.1	0.04	0.16
28	0.02	0.04	0.01	0.07	0.05	0.08	0.04	0.07	0.01	0.12	0.02	0.13	0.06	0.12	0.03	0.13	0.06	0.19
25	***0.1***	0.07	0.03	0.1	0.11	0.12	***0.05***	0.03	0.03	0.12	0.04	0.13	0.07	0.13	0.01	0.12	0.05	0.19
22	0.03	0.09	0.03	0.1	0.11	0.11	***0.9***	0.09	0.07	0.17	0.02	0.14	0.01	0.16	0.1	0.11	***0.92***	0.27
19	0.02	0.1	0.03	0.08	***0.14***	0.12	0.07	0.07	***0.31***	0.2	***0.14***	0.11	0.08	0.17	0.1	0.15	0.18	0.25

Table S6 : QF for Youden for each convergence reaching classifier for each experimental condition (cohort 2). Bold and italic values: QF1’ ≥ QF2’.

Number of patients	LR		RFELR		PCALR		ROCLR		SVM		RFESVM		PCASVM		ROCSVM		LASSO	
	QF1’	QF2’	QF1’	QF2’	QF1’	QF2’	QF1’	QF2’	QF1’	QF2’	QF1’	QF2’	QF1’	QF2’	QF1’	QF2’	QF1’	QF2’
73	0.01	0.01	0.00	0.00	0.00	0.01	***0.02***	0.01	0.01	0.01	0.00	0.01	0.00	0.01	0.01	0.02	0.01	0.04
70	0.01	0.01	0.00	0.01	0.00	0.01	***0.02***	0.01	0.00	0.00	0.01	0.01	0.01	0.01	0.01	0.02	0.01	0.04
67	0.01	0.01	0.00	0.01	0.00	0.00	0.01	0.01	0.00	0.01	0.01	0.01	0.00	0.01	0.00	0.01	0.02	0.04
64	0.01	0.01	0.00	0.00	***0.01***	0.00	0.01	0.01	0.01	0.01	0.01	0.03	0.00	0.01	0.01	0.02	0.01	0.03
61	0.01	0.01	0.00	0.01	0.01	0.01	***0.02***	0.01	0.01	0.01	0.01	0.04	0.00	0.00	0.00	0.02	0.01	0.04
58	***0.01***	0.00	0.00	0.01	0.00	0.01	0.00	0.01	0.00	0.01	0.01	0.01	0.00	0.00	0.00	0.01	0.01	0.04
55	0.01	0.01	0.00	0.00	0.00	0.01	***0.01***	0.00	0.00	0.01	0.01	0.02	0.00	0.01	0.00	0.01	0.02	0.04
52	***0.01***	0.00	0.00	0.00	0.01	0.01	0.01	0.02	0.00	0.01	0.01	0.01	0.00	0.01	0.01	0.02	0.01	0.05
49	0.01	0.01	0.00	0.01	0.01	0.01	***0.05***	0.01	0.00	0.01	0.01	0.02	0.00	0.01	0.01	0.02	0.02	0.05
46	***0.01***	0.00	0.00	0.00	0.01	0.01	***0.01***	0.00	0.00	0.01	0.01	0.01	0.00	0.02	0.01	0.03	0.01	0.05
43	***0.01***	0.00	0.00	0.01	0.01	0.01	0.01	0.01	0.00	0.01	0.01	0.03	0.00	0.02	0.01	0.02	0.02	0.06
40	***0.02***	0.00	0.01	0.01	0.01	0.01	***0.01***	0.00	0.01	0.02	0.01	0.02	0.00	0.01	0.01	0.01	0.04	0.07
37	***0.01***	0.00	0.00	0.02	0.01	0.01	***0.39***	0.01	0.01	0.01	0.01	0.05	0.01	0.02	0.01	0.02	0.03	0.07
34	***0.02***	0.00	0.00	0.01	0.01	0.02	***0.08***	0.01	0.01	0.02	0.01	0.03	0.01	0.02	0.01	0.02	0.05	0.10
31	***0.01***	0.00	0.00	0.01	0.00	0.01	0.00	0.01	0.01	0.02	0.01	0.07	0.01	0.02	0.01	0.02	0.02	0.08
28	***0.01***	0.00	0.01	0.01	0.00	0.01	0.00	0.01	0.01	0.03	0.01	0.03	0.01	0.01	0.01	0.03	0.03	0.10
25	***0.02***	0.01	0.00	0.02	0.02	0.02	0.00	0.00	0.01	0.03	0.01	0.09	0.01	0.02	0.01	0.02	0.02	0.09
22	***0.03***	0.01	0.01	0.02	0.02	0.02	0.01	0.01	0.01	0.04	0.01	0.04	0.01	0.03	0.01	0.04	***0.96***	0.14
19	0.01	0.01	0.01	0.01	0.02	0.02	0.00	0.00	0.01	0.07	0.01	0.04	0.01	0.07	0.01	0.04	0.09	0.12

Table S7 : QF for AUC for each convergence reaching classifier for each experimental condition (cohort 2). Bold and italic values: QF1’≥ QF2’.

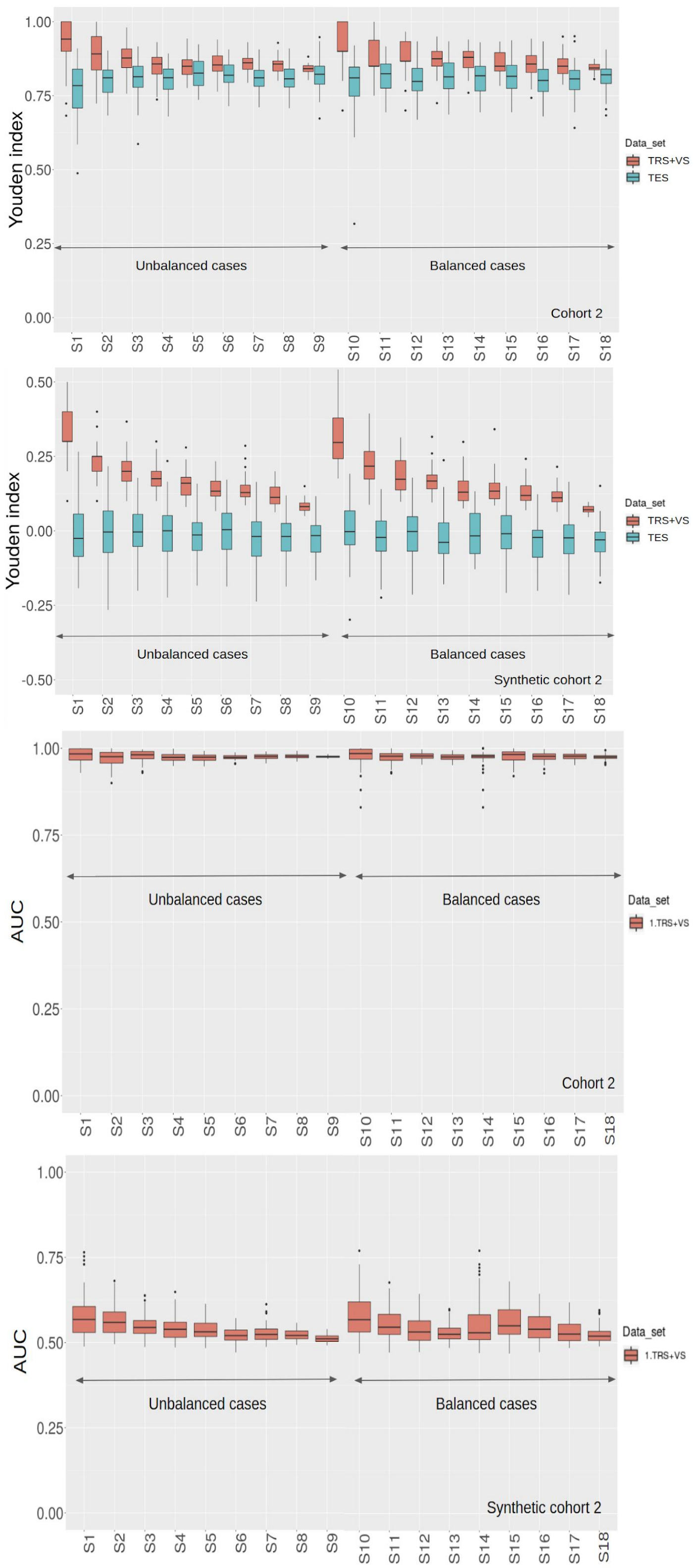

(a)

(b)

(c)

(d)

Figure S1.a: $Y_{i,ROC}^{TRS+VS}$ *(red) and* $Y_{i,ROC}^{TES}$ *(blue) for the 14 different experimental conditions of cohort 1 for the ROC study involving worst perimeter.*

S1.b: Same as 1.a for the ROC study of the synthetic cohort 2 with no predictive information involving worst perimeter.

S1.c: AUC on TRS+VS (red) and TES (blue) for the 14 different experimental conditions of cohort 1 for the ROC study involving worst perimeter.

1.d:Same as 1.c for the the synthetic cohort 2 with no predictive information involving worst perimeter.

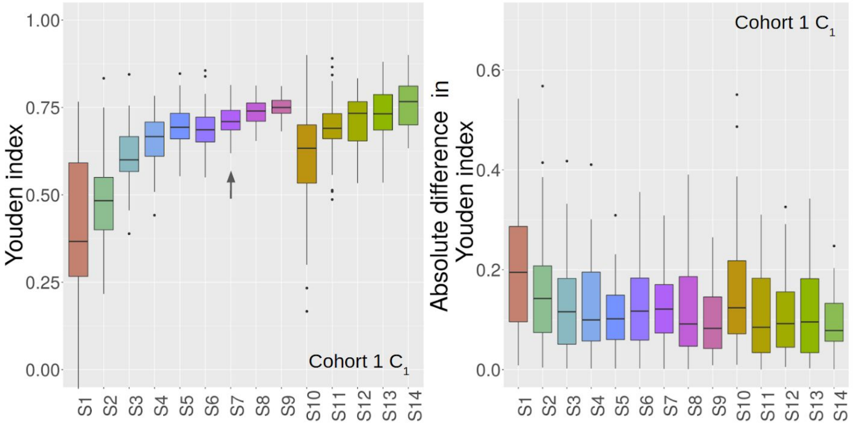

(a) *(b)*

Figure S2a: $Y^{VS}_{C_1}$ *for the 14 experimental conditions for* C_1*. ↑ shows when stability is reached for unbalanced cases (cohort 1).*

S2b. Absolute differences in Youden index between $Y^{VS}_{C_1}$ *and* $Y^{TES}_{C_1}$ *for* C_1 *(cohort 1).*

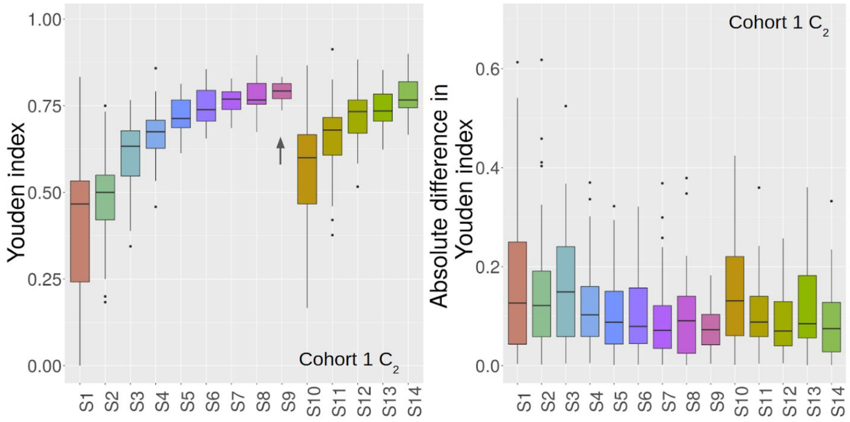

(a) *(b)*

Figure S3a: $Y^{VS}_{C_2}$ *for the 14 experimental conditions for* C_2*. ↑ shows when stability is reached for unbalanced cases (cohort 1).*

S3b. Absolute differences in Youden index between $Y^{VS}_{C_2}$ *and* $Y^{TES}_{C_2}$ *for* C_2 *(cohort 1).*

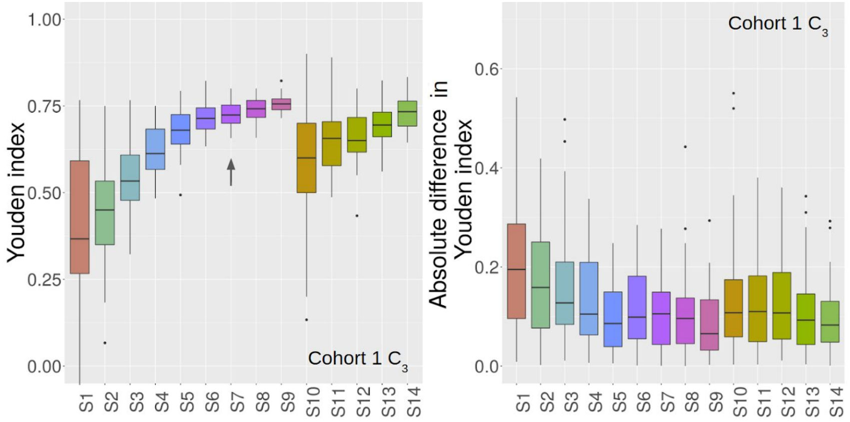

(a) *(b)*

Figure S4a: $Y^{VS}_{C_3}$ *for the 14 experimental conditions for* C_3*. ↑ shows when stability is reached for unbalanced cases (cohort 1).*

S4b. Absolute differences in Youden index between $Y^{VS}_{C_3}$ *and* $Y^{TES}_{C_3}$ *for* C_3 *(cohort 1).*

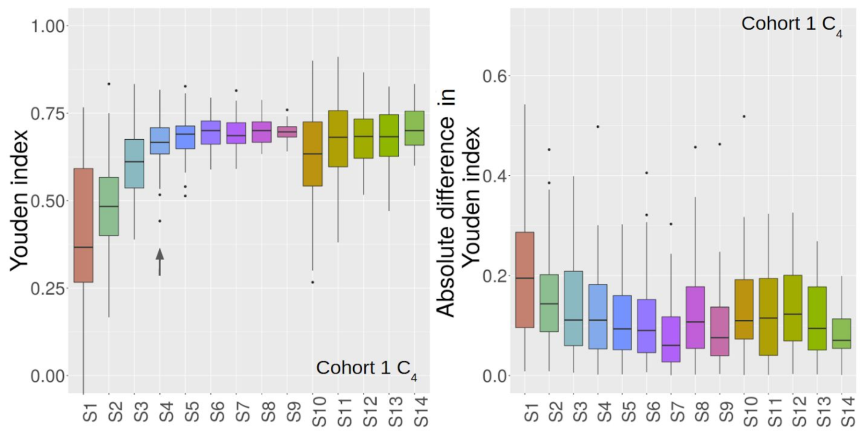

(a) *(b)*

Figure S5a: $Y_{C_4}^{VS}$ for the 14 experimental conditions for C_4. ↑ shows when stability is reached for unbalanced cases (cohort 1).
S5b. Absolute differences in Youden index between Y_4^{VS} and $Y_{C_4}^{TES}$ for C_4 (cohort 1).

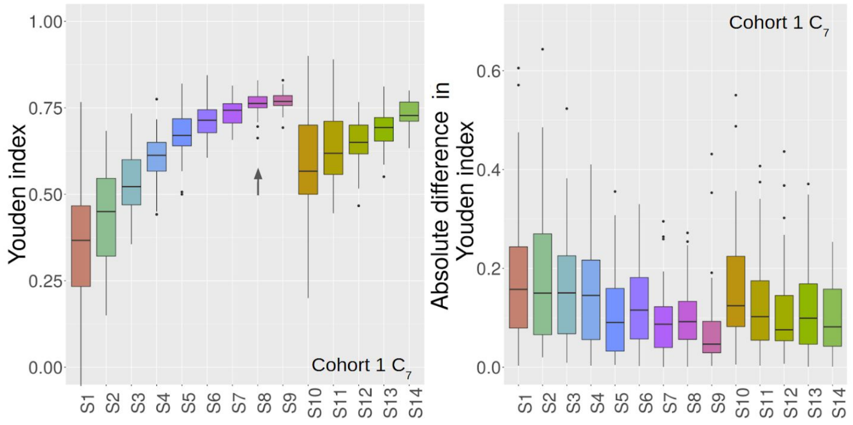

(a) *(b)*

Figure S6a: $Y_{C_7}^{VS}$ for the 14 experimental conditions for C_7. ↑ shows when stability is reached for unbalanced cases (cohort 1).
S6b. Absolute differences in Youden index between $Y_{C_7}^{VS}$ and $Y_{C_7}^{TES}$ for C_7 (cohort 1).

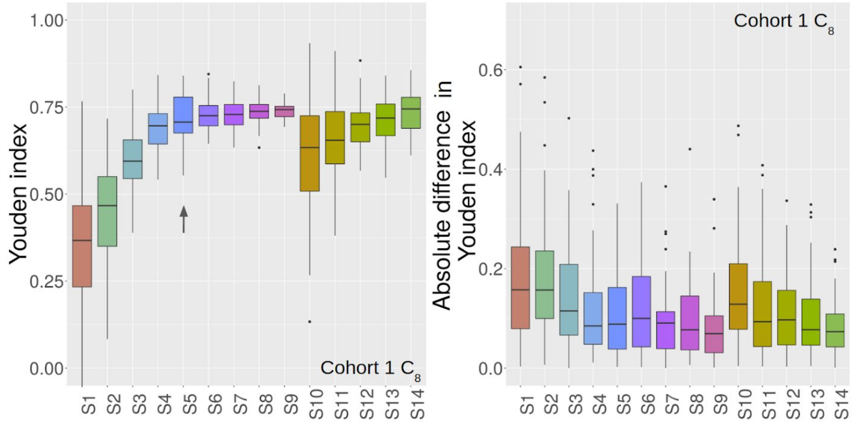

(a) *(b)*

Figure S7a: $Y_{C_8}^{VS}$ for the 14 experimental conditions for C_8. ↑ shows when stability is reached for unbalanced cases (cohort 1).
S7b. Absolute differences in Youden index between $Y_{C_8}^{VS}$ and $Y_{C_8}^{TES}$ for C_8 (cohort 1).

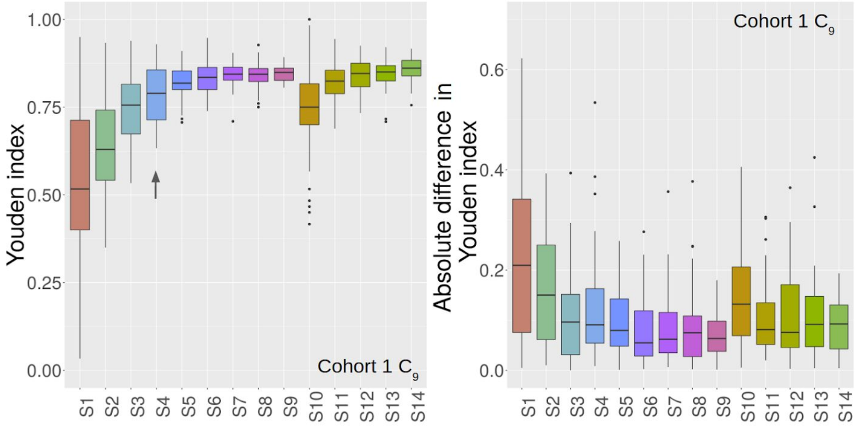

(a) *(b)*

Figure S8a: $Y_{C_9}^{VS}$ *for the 14 experimental conditions for* C_9*. ↑ shows when stability is reached for unbalanced cases (cohort 1).*

S8b. Absolute differences in Youden index between $Y_{C_9}^{VS}$ *and* $Y_{C_9}^{TES}$ *for* C_9 *(cohort 1).*

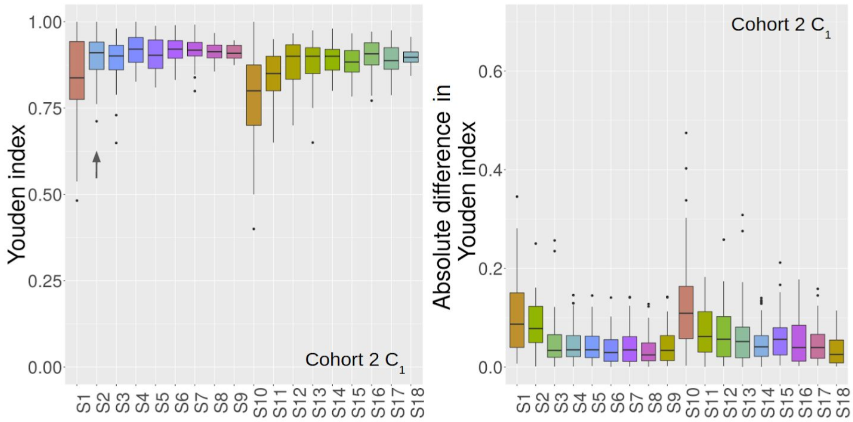

(a) *(b)*

Figure S9a: $Y_{C_1}^{VS}$ *for the 18 experimental conditions for* C_1*. ↑ shows when stability is reached for unbalanced cases (cohort 2).*

S9b. Absolute differences in Youden index between $Y_{C_1}^{VS}$ *and* $Y_{C_1}^{TES}$ *for* C_1 *(cohort 2).*

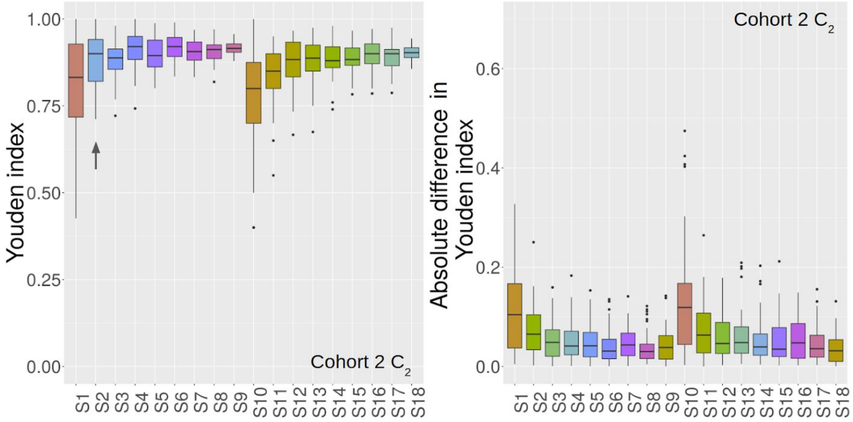

(a) *(b)*

Figure S10a: $Y_{C_2}^{VS}$ *for the 18 experimental conditions for* C_2*. ↑ shows when stability is reached for unbalanced cases (cohort 2).*

S10b. Absolute differences in Youden index between $Y_{C_2}^{VS}$ *and* $Y_{C_2}^{TES}$ *for* C_2 *(cohort 2).*

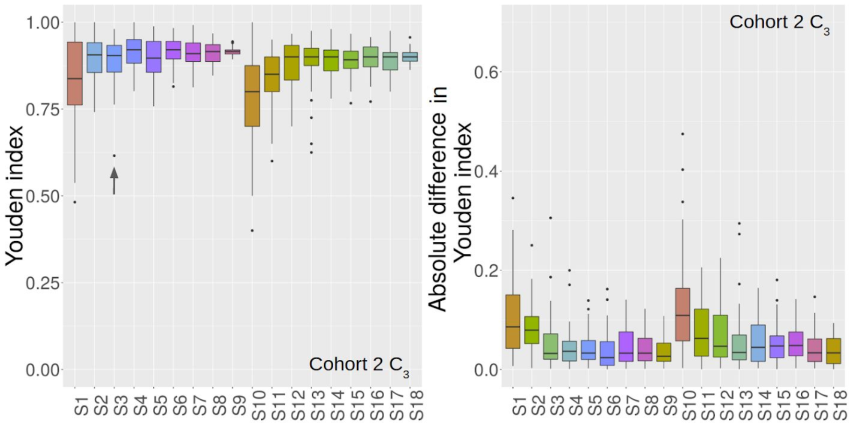

(a) *(b)*

Figure S11a: $Y_{C_3}^{VS}$ for the 18 experimental conditions for C_3. ↑ shows when stability is reached for unbalanced cases (cohort 2).

S11b. Absolute differences in Youden index between $Y_{C_3}^{VS}$ and $Y_{C_3}^{TES}$ for C_3 (cohort 2).

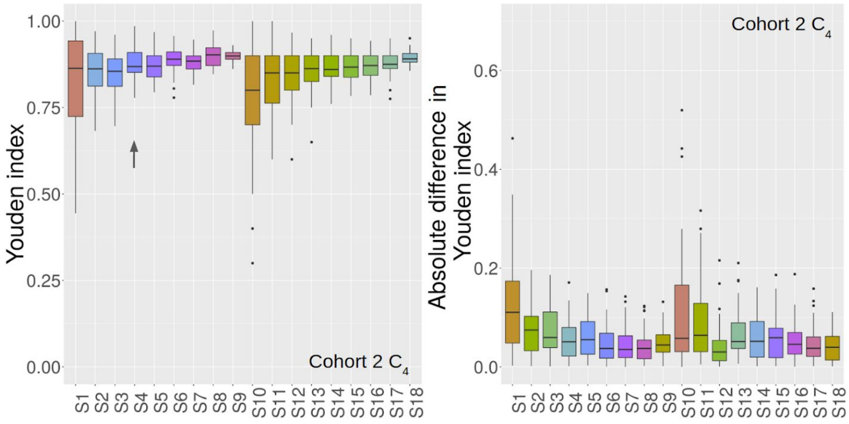

(a) *(b)*

Figure S12a: $Y_{C_4}^{VS}$ for the 18 experimental conditions for C_4. ↑ shows when stability is reached for unbalanced cases (cohort 2).

S12b. Absolute differences in Youden index between $Y_{C_4}^{VS}$ and $Y_{C_4}^{TES}$ for C_4 (cohort 2).

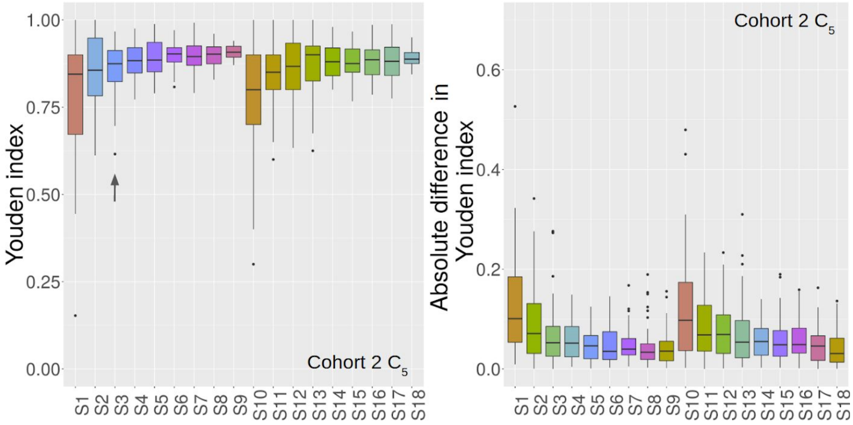

(a) *(b)*

Figure S13a: $Y_{C_5}^{VS}$ for the 18 experimental conditions for C_5. ↑ shows when stability is reached for unbalanced cases (cohort 2).

S13b. Absolute differences in Youden index between $Y_{C_5}^{VS}$ and $Y_{C_5}^{TES}$ for C_5 (cohort 2).

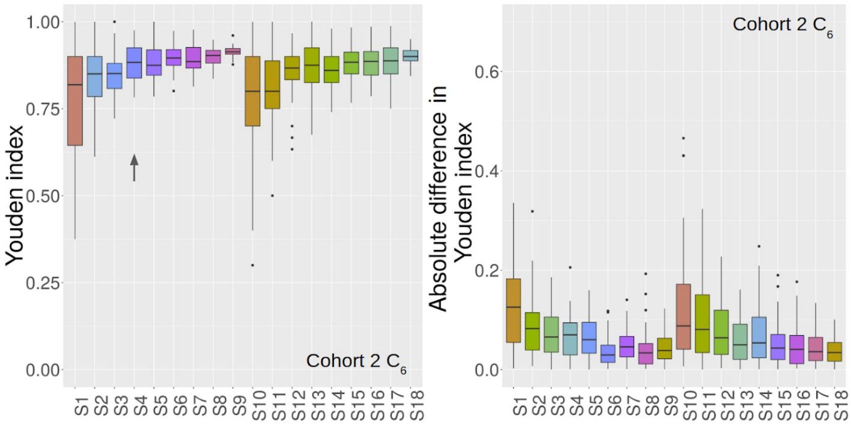

(a) *(b)*

Figure S14a: $Y_{C_6}^{VS}$ for the 18 experimental conditions for C_6. ↑ shows when stability is reached for unbalanced cases (cohort 2).
S14b. Absolute differences in Youden index between $Y_{C_6}^{VS}$ and $Y_{C_6}^{TES}$ for C_6 (cohort 2).

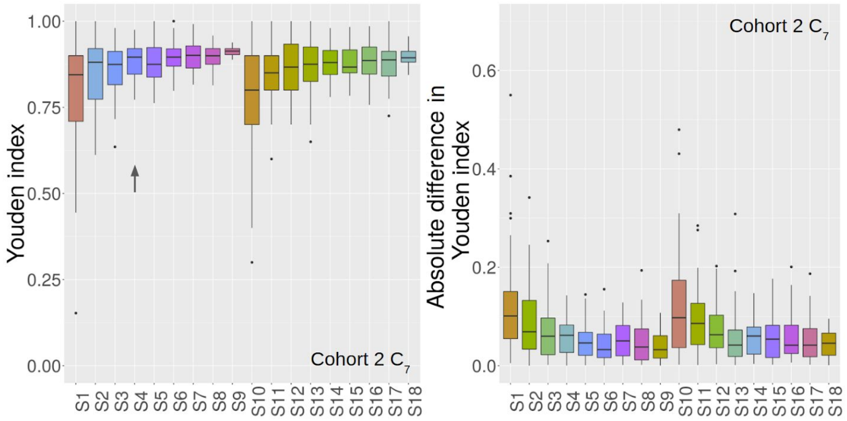

(a) *(b)*

Figure S15a: $Y_{C_7}^{VS}$ for the 18 experimental conditions for C_7. ↑ shows when stability is reached for unbalanced cases (cohort 2).
S15b. Absolute differences in Youden index between $Y_{C_7}^{VS}$ and $Y_{C_7}^{TES}$ for C_7 (cohort 2).

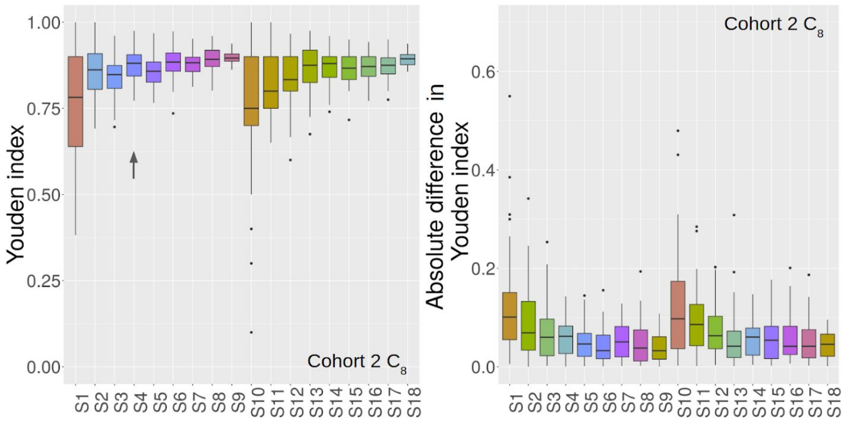

(a) *(b)*

Figure S16a: $Y_{C_8}^{VS}$ for the 18 experimental conditions for C_8 (cohort 2). ↑ shows when stability is reached for unbalanced cases (cohort 2).
S16b. Absolute differences in Youden index between $Y_{C_8}^{VS}$ and $Y_{C_8}^{TES}$ for C_8 (cohort 2).

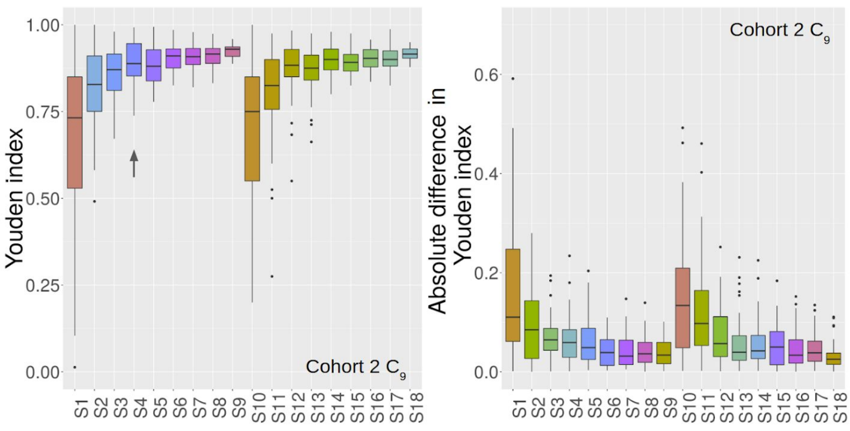

(a) *(b)*

Figure S17a: $Y_{C_9}^{VS}$ *for the 18 experimental conditions for* C_9*.* ↑ *shows when stability is reached for unbalanced cases (cohort 2).*

S17b. Absolute differences in Youden index between $Y_{C_9}^{VS}$ *and* $Y_{C_9}^{TES}$ *for* C_9 *(cohort 2).*
